# Using Multiple Methods to Estimate Respiratory Syncytial Virus (RSV)‐associated Hospitalization Rates in Children Aged < 5 Years—Hamilton County, Ohio, 2009–2017

**DOI:** 10.1111/irv.70096

**Published:** 2025-04-16

**Authors:** Elizabeth J. Harker, Ryan Wiegand, Erica Billig Rose, Marilyn Rice, Christina Quigley, Chelsea Rohlfs, Susan I. Gerber, Gayle E. Langley, Heidi L. Moline, Mary Allen Staat, Meredith L. McMorrow

**Affiliations:** ^1^ National Center for Immunization and Respiratory Diseases Centers for Disease Control and Prevention Atlanta Georgia USA; ^2^ US Public Health Service Rockville Maryland USA; ^3^ Division of Infectious Diseases Cincinnati Children's Hospital Medical Center Cincinnati Ohio USA; ^4^ Department of Pediatrics University of Cincinnati Cincinnati Ohio USA

**Keywords:** burden estimates, capture–recapture analysis, respiratory syncytial virus

## Abstract

**Background:**

Respiratory syncytial virus (RSV) is a leading cause of lower respiratory tract infection in children less than 5 years of age worldwide. In the United States, RSV commonly causes hospitalization in young children and is the leading cause of hospitalizations in infants. As new RSV immunizations become available, burden estimates are critical to guide the implementation of recommendations and quantify impact.

**Methods:**

We estimated RSV‐associated hospitalization rates at a large US pediatric medical center during an 8‐year period using five approaches, namely, estimation directly from active and passive surveillance systems, both a crude and stratified capture–recapture analysis of data from both systems, and estimation based on discharge diagnosis codes. The stratified analysis was performed to ensure adherence with the capture–recapture methodology assumption that samples are independent and participants have an equal probability of being observed within each system.

**Results:**

Overall, estimated RSV‐associated hospitalization rates per 1000 children were 4.0 (2.5, 6.1) based on adjusted estimates from active surveillance, 1.7 (2.1, 4.4) from passive surveillance, 7.9 (5.7, 13.0) from crude capture–recapture analysis, 5.0 (3.8, 7.2) from the stratified capture–recapture, and 4.4 (4.0, 4.9) from discharge diagnosis codes.

**Conclusions:**

Each method has limitations and inherent biases that may impact the estimation of the burden of RSV. Capture–recapture analysis may be a useful tool to estimate the burden of RSV, but needs to be adjusted to account for possible violation of the assumptions of independence and equal probability of capture to ensure accurate approximation of disease burden and avoid over estimation.

## 1 Background

Respiratory syncytial virus (RSV) is a leading cause of lower respiratory tract infection in children less than 5 years of age worldwide. Globally, RSV is estimated to cause more than 3 million hospitalizations and between 66,000 and 199,000 deaths, with 20% of hospitalized children with acute respiratory infections (ARIs) infected with RSV [1, 2, 3, 4]. In the United States, RSV commonly causes hospitalization in young children and is the leading cause of hospitalizations in infants, with previous studies finding the highest rate of infection among infants less than 2 months of age (25.9 per 1000 children) [3, 5]. In 2023, new RSV immunizations to prevent RSV in infants and young children became available, including a vaccine for pregnant women and a monoclonal antibody for infants and some young children [6, 7]. Additional pediatric immunizations are in development. As new RSV immunizations become available, burden estimates are critical to guide immunization recommendations and quantify their impact [8].

Population‐based rates of RSV‐associated hospitalization in children less than 5 years of age have been estimated using active, prospective surveillance of acute respiratory infections [3, 9]. Active surveillance that includes systematic collection of respiratory specimens and laboratory testing for RSV has the advantage of capturing infection cases that may be missed by clinician‐directed testing. However, estimates based on active surveillance must be adjusted for nonenrollment and nonsurveillance days. Maintaining systematic, high‐quality active surveillance also may have higher financial and personnel costs than passive laboratory‐based surveillance of clinical test results.

Alternative methods have been used to estimate population‐based hospitalization rates, including indirect analyses using discharge diagnosis codes [4, 10]. Although these methods are less costly, further investigation is needed to understand whether these methods can be used to accurately estimate RSV‐associated hospitalizations, because patients hospitalized with pneumonia and bronchiolitis may have nonpathogen‐specific discharge diagnosis codes, and the percentage of these associated with RSV must be estimated.

Capture–recapture analysis is a popular methodology to account for imperfect surveillance in epidemiological studies [11, 12, 13, 14, 15, 16, 17, 18]. Capture–recapture analysis requires a closed‐population with at least two independent surveillance systems, and patients must have equal probability of being observed within each system, although the probabilities may differ between the two systems. Assuming that patients can be uniquely identified and matched between surveillance system, the number of patients matched and unmatched in each surveillance system can be used to estimate the total number of patients observed and unobserved within each surveillance system, including the number of patients missed by both surveillance systems, and the overall rate of hospitalization.

We estimated RSV‐associated hospitalization rates at a large US pediatric medical center during an 8‐year period using five approaches, including estimation directly from active and passive surveillance systems, capture–recapture analysis of data from both systems, and estimation based on discharge diagnosis codes.

## 2 Methods

### 2.1 Study Site

Cincinnati Children's Hospital Medical Center (CCHMC), a 762‐bed academic pediatric medical center, is the only children's hospital in Hamilton County, and cares for more than 95% of Hamilton County children requiring hospitalization. More than 1 million patient encounters occur annually at the primary hospital (Burnet Campus) surveillance site including > 28,000 inpatient hospitalizations.

### 2.2 Active Surveillance System

Active surveillance was conducted at CCHMC during eight surveillance years (July 1, 2009–June 30, 2017) for an average of 2–5 days per week, with only those enrolled on surveillance days used in estimations. During the first five surveillance years (2009–2010, 2010–2011, 2011–2012, 2012–2013, and 2013–2014), active surveillance was conducted an average of 3.25 days per week, compared with an average of 4.8 days per week during the last three surveillance years. The Epidemiology and Surveillance Program in the Division of Infectious Diseases at CCHMC enrolled children aged less than 5 years who resided in Hamilton County at the time of hospital admission and presented with fever and/or acute respiratory symptoms (Table S5). Children were excluded if they had neutropenia or were a newborn and never left the hospital. In addition, children whose parents or guardians refused consent or were unable to understand the consent process, including those with a language barrier, were not eligible. To ensure cases were community acquired, hospitalized patients admitted for more than 48 h or who were enrolled as outpatients within the previous 6 days were excluded. Nasal and oropharyngeal (OP) specimens (2009–2014) and mid‐turbinate nasal and OP specimens (2015–2017) were collected from all eligible patients. Specimens were collected from each enrolled child within 14 days of symptom onset and were tested using a multiplex PCR (Luminex) [19].

For cases that were captured by passive surveillance and not active surveillance, medical record abstraction was conducted to determine if there were missed due being admitted on a nonsurveillance day, were not eligible for enrollment, were eligible but not enrolled, or were missed by enrollers.

### 2.3 Passive Surveillance System

CCHMC monitors the prevalence of infectious diseases through a passive, clinical laboratory surveillance system which can identify hospitalized children who had a positive RSV test result as part of their usual medical care. From 2009 to 2017, 77% of tests performed were PCR tests, while 23% were antigen tests. Children aged less than 5 years were included in this analysis if they were residents of Hamilton County, Ohio, were hospitalized at CCHMC during the study period and had RSV detected from a respiratory specimen collected as part of routine care within 14 days of hospital admission.

Medical record abstraction was conducted for those who were captured through active surveillance and not passive surveillance to determine if they were missed due to not being tested for RSV. Cases also could have been missed if they tested negative through routine laboratory testing and positive through research testing performed as part of active surveillance.

### 2.4 Denominator Data

Census estimates of children aged less than 5 years in Hamilton County, Ohio, on July 1 were used as denominators for rates in each surveillance year [20] (except for 2009 which was extrapolated using a simple linear regression fit to census estimates for 2010–2017).

### 2.5 Capture–Recapture Methodology

Data from both the passive and active surveillance systems were collected from July 1, 2009, to June 30, 2017. For each surveillance year (July 1–June 30), cases identified through the active surveillance were matched to those identified through passive surveillance. Children were matched by medical record number, date of birth, and date of hospitalization. Children identified by both systems with hospitalization dates within 14 days of each other were considered a match. Multiple hospitalizations of one patient within 14 days were considered the same event.

We conducted an initial crude capture–recapture analysis using Petersen's capture–recapture methodology with Evans' small sample size adjustment^1^ to estimate the total number of children hospitalized for RSV using the cases reported from both surveillance systems and matched cases [21]. Confidence intervals (CIs) were calculated using bias‐corrected and accelerated bootstraps [22].

We then performed a stratified analysis restricted to both active and passive participants admitted on active surveillance enrollment days. Because the capture–recapture methodology assumes participants have an equal probability of being observed within each system, limiting by day of enrollment ensured the possibility of being captured by the active system. We stratified the analysis by variables that may have influenced the probability of capture in one or both systems (race, age, and ICU status). We then totaled the estimated numbers of cases to estimate the total number of hospitalizations and then overall RSV‐associated hospitalization rates [18].

### 2.6 Adjusted Active Surveillance Rate Estimation

Adjusted hospitalization rates were calculated from active surveillance, dividing the number of hospitalizations meeting ARI case definition identified through active surveillance enrollment by the estimated population denominator from census data as described above. Adjustments were made to account for nonenrollment by multiplying rates by the inverse of proportion of children enrolled out of those who were eligible for active surveillance, and by the inverse of proportion of days per week surveillance was conducted, plus an extra day to further account for days missed.

### 2.7 Discharge Diagnosis Code Analysis

Hospital discharge diagnosis codes were abstracted for all children hospitalized at CCHMC who were < 5 years of age and residents of Hamilton County during the study period. For this analysis, discharge diagnosis codes of interest included RSV‐specific disease (RSV bronchiolitis, RSV pneumonia, and RSV as a cause of disease classified elsewhere), unspecified bronchiolitis, and pneumonia not due to RSV (Table S1). During July 1, 2009–September 30, 2015, ICD‐9 codes were used, and during October 1, 2015–June 30, 2017, ICD‐10 codes were used with a period of overlap during transition.^2^ To estimate RSV‐associated hospitalizations, we included all RSV‐specific diagnosis codes, 30% of the unspecified bronchiolitis codes, and 20% of the pneumonia codes not coded as RSV that were recorded during November through April of each surveillance year. These percentages were derived from New Vaccine Surveillance Network (NVSN) data [7]. Hospitalization rates were estimated by dividing total RSV‐associated hospitalizations estimated from discharge diagnosis codes by census population denominators. Ninety‐five percent CIs were calculated using bias‐corrected and accelerated bootstraps [22]. These results were compared with results from the capture–recapture, adjusted active, and passive surveillance estimates.

Estimated rates were considered significantly different if 95% CIs did not overlap.

All analyses were done in R‐3.4.1. This activity was reviewed by CDC and was conducted consistent with applicable federal law and CDC policy.^3^

## 3 Results

### 3.1 Active and Passive Surveillance Systems

**FIGURE 1:**
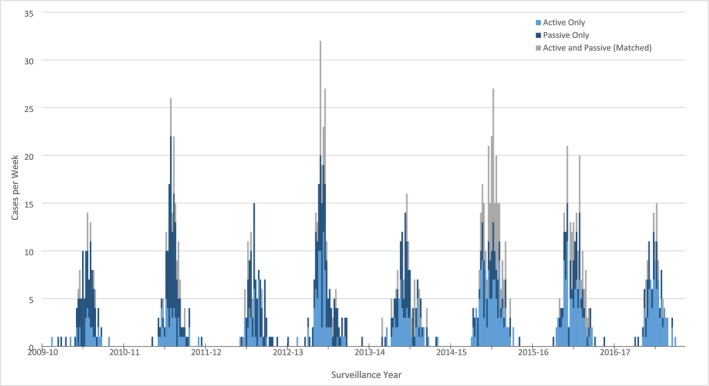
Case count per week during each surveillance year, July 1 – June 30, 2009–2017. Major tick marks indicate start of surveillance year, minor tick marks indicate January of year.

Overall, 1257 cases of laboratory‐confirmed RSV‐associated hospitalization were observed in the active and passive surveillance systems between July 1, 2009, and June 30, 2017. Of these, 154 were captured by both surveillance systems, 583 were captured by passive surveillance but missed by active surveillance, and 520 were captured by active surveillance but missed by passive surveillance. Both active and passive surveillance captured patients throughout the RSV season, and RSV seasonality was consistent across surveillance years (Figure 1). RSV‐associated hospitalizations were common during November through April, peaking in January and February.

### 3.2 Reasons for Missed RSV Hospitalizations

Of the 520 patients missed by passive surveillance, 505 (97%) were missed because they were not tested for RSV as part of routine clinical care (Table S3). Fifteen (3%) patients were tested for RSV, but the test was negative by routine laboratory testing and positive in research testing in active surveillance. Of the 583 patients missed by active surveillance, 324 (56%) were admitted on a nonsurveillance day, 41 (7%) were not eligible for enrollment, 143 (18%) were eligible but not enrolled, and 75 (13%) were not screened by surveillance staff.

### 3.3 Capture–Recapture Analysis

Using the crude capture–recapture methodology, we estimated 3409 hospitalizations meaning 2152 cases (63% of total cases) were missed by both surveillance systems while the stratified capture–recapture analysis estimated 2134 hospitalizations with 877 cases (41% of total cases) missed.

**FIGURE 2:**
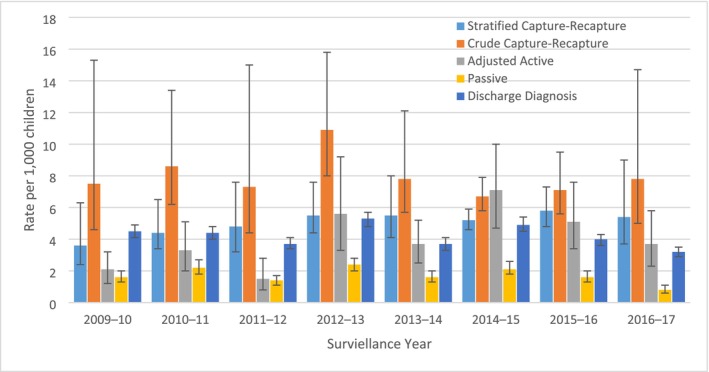
Comparison of estimated RSV‐associated hospitalization rates per 1000 among children less than 5 years of age using five methods, Hamilton County, July 1–June 30, 2009–2017. Stratified capture–recapture was conducted using Petersen's capture–recapture methodology with Evans' small sample size adjustment. Analysis limited to those admitted on enrollment days. Analysis variables that may have influenced the probability of capture in one or both systems (race, age, and ICU status) then numbers of hospitalizations from all strata were totaled to estimate the overall RSV‐associated hospitalization rates. Confidence intervals calculated using 10,000 bias‐corrected and accelerated bootstrap replicates. Crude capture–recapture was conducted using Petersen's capture–recapture methodology with Evans' small sample size adjustment. Confidence intervals calculated using bias‐corrected and accelerated bootstrap replicates. Adjusted active rates were adjusted to account for nonenrollment by multiplying rates by the inverse of proportion of children enrolled out of those who were eligible, and by the inverse of proportion of days per week surveillance was conducted plus one extra day to account for missing days. Confidence intervals calculated using bias‐corrected and accelerated bootstrap replicates. Passive surveillance rates were estimated by dividing the number of hospitalizations identified through passive surveillance by the estimated population denominator. Poisson confidence intervals were used. Discharge diagnosis estimates were calculated using all RSV‐specific coded hospitalizations as well as 20% of unspecified pneumonia and 30% of unspecified bronchiolitis codes from patients admitted from November to April. Confidence intervals calculated using 10,000 bias‐corrected and accelerated bootstrap replicates. Numbers for graph are given in Table S1.

The average RSV‐associated hospitalization rate using the crude capture–recapture methods across all 8 years was 7.9 (95% CI: 5.7, 13.0) per 1000 children (Table S1). By season, hospitalization rates ranged from 6.7 (95% CI: 5.8, 7.9) during 2014–15 to 10.9 (95% CI: 8.0, 15.8) per 1000 children during 2013–2014 (Figure 2). Compared with the crude estimates, rates estimated by stratified capture–recapture analysis were lower with an overall RSV‐associated hospitalization rate of 5.0 (95% CI: 3.8, 7.2) per 1000 children per surveillance year. Estimated hospitalization rates from the stratified analysis varied by surveillance year, ranging from 3.5 (95% CI: 2.3, 6.2) during 2009–2010 to 5.8 (95% CI: 4.8, 7.3) during 2015–2016 (Table 1).

**TABLE 1:** Estimated number and rate per 1000 children of RSV‐associated hospitalizations among children < 5 years of age based on stratified capture–recapture analysis^a^ of cases identified by active and passive, Hamilton County, Ohio, 2009–2017.

Surveillance year	Matched (*N*)	Passive only (*N*)	Active only (*N*)	Estimated number of hospitalizations	Population^b^	Estimated rate per 1000 (95% CI^c^)
2009–10	7	33	26	184.4	53,047	3.5 (2.3, 6.2)
2010–11	15	45	44	234.6	53,187	4.4 (3.4, 6.5)
2011–12	7	42	31	258.5	53,378	4.8 (3.2, 7.6)
2012–13	20	44	71	290.2	53,306	5.4 (4.3, 7.5)
2013–14	16	46	61	296.3	53,415	5.5 (4.1, 8.0)
2014–15	53	37	108	274.0	53,592	5.1 (4.5, 5.8)
2015–16	27	43	94	313.5	54,192	5.8 (4.8, 7.3)
2016–17	9	21	77	282.4	53,694	5.3 (3.6, 8.9)
Overall	154	311	512	2133.9	427,811	5.0 (3.8, 7.2)

^a^ Analysis limited to those admitted on enrollment days. Analysis stratified by variables that may have influenced the probability of capture in one or both systems (race, age, and ICU status) then numbers of hospitalizations from all strata were totaled to estimate the overall RSV‐associated hospitalization rates. Analysis used Evans' small sample size adjustment.

^b^ US census estimates of children aged less than 5 years in Hamilton County, Ohio.

^c^ Confidence intervals calculated using 10,000 bias‐corrected and accelerated bootstrap replicates.

Hospitalization rates also varied by age category. Children aged less than 6 months had the highest rates of RSV‐associated hospitalization, with the highest stratified rate seen among those aged 2 to 3 months, at 24.9 per 1000 children (95% CI: 20.5, 32.1) (Table 2). Stratified RSV‐associated hospitalization rates among children aged 6–11, 12–23, and 24–59 months were significantly lower at 7.5 (95% CI: 6.2, 9.6), 4.0 (95% CI: 3.3, 5.2), and 1.9 (95% CI: 1.4, 2.8), respectively. In the crude analysis, those less than 2 months of age had the highest rate of hospitalization at 37.7 (95% CI: 32.5, 45.2) while those aged 24–59 months again had the lowest rate at 2.9 (95% CI: 1.9, 4.6) (Table 2).

**TABLE 2:** Estimated number and rate per 1000 children of RSV‐associated hospitalizations stratified by age group among children < 5 years of age based on crude and stratified capture–recapture analysis^a^ of cases identified by active and passive, Hamilton County, Ohio, 2009–2017.

Age group	Matches (*N*)	Passive only (*N*)	Active only (*N*)	Population^b^	Estimated number of hospitalizations	Rate per 1000 (95% CI)^c^
Crude capture–recapture						
Less than 2 months	53	167	77	13,996	527.6	37.7 (32.5 45.2)
2 to 3 months	26	89	83	13,996	466.1	33.3 (26.2 45.2)
4 to 5 months	12	56	76	13,996	459.1	32.8 (22.6 53.8)
6 to 11 months	26	87	101	41,988	550.0	13.1 (10.1 17.8)
12 to 23 months	23	88	102	85,392	597.7	7.0 (5.3 9.8)
24 to 59 months	14	96	81	256,088	742.6	2.9 (1.9 4.6)
Overall	154	583	520	411,460	3343.1	7.9 (5.7, 13.0)
Stratified capture–recapture						
Less than 2 months	53	84	74	13,996	328.7	23.4 (20.7, 27.2)
2 to 3 months	26	59	81	13,996	349.0	24.9 (20.5, 32.1)
4 to 5 months	12	29	75	13,996	294.2	21.0 (16.0, 29.9)
6 to 11 months	26	39	100	41,988	314.8	7.5 (6.2, 9.6)
12 to 23 months	23	41	101	85,392	344.2	4.0 (3.3, 5.2)
24 to 59 months	14	59	81	256,088	488.4	1.9 (1.4,2.8)
Overall	154	311	512	411,460	2133.9	5.0 (3.8, 7.2)

^a^ Analysis limited to those admitted on enrollment days. Analysis stratified by variables that may have influenced the probability of capture in one or both systems (race, and ICU status) then numbers of hospitalizations from all strata were totaled to estimate the overall RSV‐associated hospitalization rates. Analysis used Evans' small sample size adjustment.

^b^ US census estimates with age groups for children aged less than 5 years in Hamilton County, Ohio, multiplied by 8 to account for all years of study.

^c^ Confidence intervals calculated using 10,000 bias‐corrected and accelerated bootstrap replicates.

### 3.4 Adjusted Active Surveillance Rate Estimation

Using adjusted active surveillance the average annual estimated hospitalization rate per 1000 children < 5 was 4.0 (95% CI: 2.5, 6.1). Estimates ranged from 1.5 (95% CI: 0.8, 2.8) in the 2011–12 season to 7.1 (4.7, 10.0) in the 2014–2015 season (Figure 2, Table S1).

### 3.5 Discharge Diagnosis Code Analysis

The RSV‐associated hospitalization rate per 1000 children < 5 estimated from ICD discharge diagnosis codes was 4.2 (95% CI: 3.8, 4.6) overall and ranged from 3.2 (95% CI: 2.9, 3.5) during 2016–2017, to 5.3 (95% CI: 4.8, 5.7) in the 2012–2013 season (Figure 2, Table S1).

### 3.6 Comparison of Rates by Estimation Approach

The crude capture–recapture estimated RSV‐associated hospitalization rates were higher than the passive rates during all surveillance years and higher than the adjusted active rates for all years except 2014–2015 (Figure 2, Table S1). The stratified analysis rates were higher than the passive rates during all years and the adjusted active rates during all years except 2012–2013 and 2014–2015. Furthermore, the crude capture–recapture rate was statistically significantly higher than the adjusted active rates for half of the surveillance years: 2009–2010, 2010–2011, 2011–2012, and 2013–2014. However, the stratified analysis was significantly higher than the adjusted active rate only in 2011–2012 (Figure 2, Table S1).

Rates estimated using adjusted active surveillance were consistently higher than passive surveillance estimates and were statistically significantly higher from the 2013–2014 season onward, when active surveillance was conducted four or more days per week (Figure 2, Table S1).

Estimated rates through ICD code analysis were lower than rates calculated through the crude capture–recapture analysis. However, these rates had overlapping CIs with the stratified capture–recapture estimates except for the 2015–2016 and 2016–2017 seasons. The rates estimated through discharge diagnosis codes were also significantly higher than the passive rates for every year but were only significantly higher than the adjusted active estimates in the 2009–2010 and 2011–2012 seasons, when active surveillance was conducted fewer days per week than in the last 3 years of surveillance (an average of 3.25 vs. 4.8 days a weeks) (Figure 2, Table S1).

## 4 Conclusions

This analysis uses five unique approaches to estimation to examine 8 years of data from a single large hospital. We found that the stratified capture–recapture analysis estimated RSV‐associated hospitalization rates were similar to estimates from adjusted active surveillance and to those previously reported, as well as those calculated using discharge diagnosis codes with adjustments for portion RSV‐positive [3]. There are three primary assumptions to the capture–recapture approach: (1) that the population under study is closed, (2) that the two samples are independent, and (3) that individuals in a population have similar probability of enrollment in both systems. Although our two surveillance systems did limit to the same closed population (children aged less than 5 years hospitalized at CCHMC and resident in Hamilton County), children did not have equal probability of being enrolled in the two systems. When multiple types of surveillance are available, capture–recapture methodologies may be a useful tool to account for incomplete surveillance but may also be influenced by differences in surveillance enrollment procedures. Due to this, capture–recapture analyses should be adjusted to account for differences in the populations captured within each system to provide the best approximation of burden. We found that subjects did not have an equal probability of capture within each surveillance system. Without adjusting for days in which active surveillance was not conducted, increased likelihood of clinical testing in ICU, and increased clinical testing in younger infants, application of capture–recapture methodology to hospital‐based surveillance may lead to falsely elevated estimates of RSV burden [18].

Using active surveillance, RSV‐associated hospitalization rates in Hamilton County, Ohio, were previously estimated during the 2003–2004 season to be 5.7 per 1000 children < 5 years of age and 33.9 per 1000 children aged less than 6 months [3]. This overall rate was higher but not statistically different from stratified capture–recapture estimates during 2009–2017. However, the stratified capture–recapture estimates for children aged less than 6 months during 2009–2017 are lower than this earlier estimate.

During our study period, the number of surveillance days conducted per week ranged from an average of 3.25 to 4.8 days per week. Estimates from active surveillance were closer to the capture–recapture and hospital discharge coding estimates during years when surveillance was conducted at least 4 days a week suggesting that more active surveillance days may produce more consistent estimates. However, although enrolling patients in active surveillance on more days can lead more reliable estimations, active surveillance is costly to maintain.

The most common reasons patients were missed by active surveillance were admission on a nonenrollment day and failure to enroll eligible subjects. These were accounted for in the adjustments to active surveillance analysis. Other reasons for missed patients (lack of screening, etc.) were not adjusted for and need to be evaluated further. Ninety‐seven percent of patients were missed by passive surveillance because they were not tested as part of routine clinical care. The American Academy of Pediatrics does not recommend testing for RSV in patients with bronchiolitis, the most common syndrome associated with RSV in young children [23]. Laboratory surveillance may be a cost‐effective way to monitor RSV seasonality, but because patients with respiratory illness are frequently not tested for RSV, does not adequately capture the burden of disease. Consistent testing of patients for RSV could increase those captured by passive surveillance, making this method more viable as a surveillance option.

Using discharge diagnosis codes with adjustment for percentage of RSV‐positive to estimate hospitalization rates provided rates that were not significantly different from those estimated through adjusted‐active surveillance, except in years of lower active surveillance days. This estimate utilized proportions of pneumonia and bronchiolitis cases seen in the literature and in data provided by the NVSN; however, the true percentage of these cases due to RSV is unknown [7]. Additionally, it is yet unknown what effect the introduction of RSV prevention products in young children will have on the estimated proportions of these ICD‐10 codes that can attributed to RSV. Further active surveillance is needed to accurately adjust these proportions to inform ICD code analysis. Variability in the seasonality of disease and changes in coding practices could also lead to either an overestimation or underestimation of RSV‐associated hospitalization rates. While estimated RSV‐associated hospitalizations using discharge diagnosis codes were similar than those estimated through adjusted‐active surveillance, the latter also estimated hospitalizations greater than those found through RSV alone as a discharge diagnosis (Table S2). This could indicate that a diagnosis of RSV may not accurately capture the burden of disease.

This study has at least five limitations. First, eligibility and exclusion criteria for the active surveillance system changed during the study period (Table S5). In 2015, CCHMC joined the NVSN, which identifies children within a catchment area who have ARI and test positive for RSV, within the initial days of admission. Children enrolled in NVSN were used as the active surveillance portion of the study. Although changes were minimal, differences may have affected which patients were captured by surveillance year. Second, although we attempted to account for all differences within the active versus passive surveillance systems populations using the stratified analysis, it is possible that there are other unknown variables that influenced the probability of a subject being captured through one system versus the other. To our knowledge, methods for identifying appropriate variables for the stratification analysis are not generally agreed upon, and variables that we used may not be optimal. Third, the method of adjusting for days when surveillance was done does not account for possible day‐of‐week differences in mean number of admissions. Fourth, our study included residents of Hamilton County, Ohio, and may not be generalizable to all US children. Fifth, although CCHMC cares for more than 95% of children in Hamilton Country, it is possible that some children who reside in the county could have been hospitalized for RSV at a different hospital.

We compared five RSV hospitalization surveillance methods and found that hospitalization rates for three were similar to those previously reported for a prior period using an active surveillance system, while two were dissimilar. However, each method has limitations and inherent biases that may impact the estimation of the burden of RSV within the area. Without further adjustment, capture–recapture analysis can lead to an overestimation of the burden of disease. However, in instances where active surveillance cannot be conducted regularly, capture–recapture analysis may be a useful tool to estimate the burden of RSV, as long as adjustments are performed to account for the assumption of equal probability of capture by both systems.

## Author Contributions

Elizabeth J. Harker was the primary writer of this manuscript as well as main epidemiologist conducting analyses. Ryan Wiegand provided technical and statistical support for the methodology and coding of the capture–recapture analyses. Erica Billig Rose began the initial analyses and manuscript and conducted the adjusted active‐surveillance portion. Marilyn Rice, Christina Quigley, and Chelsea Rohlfs all provided support at CCHMC to collect the initial data, as well as cleaning and organizing these data for CDC. Susan I. Gerber and Gayle E. Langley aided in the initial conceptualization of this manuscript. Heidi L. Moline managed the NVSN project and provided support creating the manuscript. May Allen Staat is a senior author who oversaw the collection of data from CCHMC and aided in creating the initial study. Meredith L. McMorrow is a senior author who provided methodology support and oversaw the CDC NVSN team. All authors reviewed and provided feedback on the final manuscript.

## Disclosure

The findings and conclusions in this report are those of the authors and do not necessarily represent the official position of the Centers for Disease Control and Prevention.

## Ethics Statement

This work was conducted with the approval of both the Cincinnati Children's Hospital Medical Center IRB and the Centers for Disease Control and Prevention IRB.

## Consent

For active surveillance consent was required for participants' information to be collected. Consent was not needed for passive surveillance or ICD code review.

## Conflicts of Interest

Cincinnati Children's Hospital Medical Center, where these data were collected and with whom several authors are affiliated has received funding from GlaxoSmithKline, Cepheid, and MERCK. Additionally, Susan Gerber is now employed at GlaxoSmithKline though she was employed at CDC during her contribution to this manuscript. There are no other known conflicts of interest.

### Peer Review

The peer review history for this article is available at https://www.webofscience.com/api/gateway/wos/peer‐review/10.1111/irv.70096.

## Permission to Reproduce Material From Other Sources

Any interested party has permission to reproduce this material from other sources.

## Supporting information


**Table S1.** Estimated Number and Rate per 1000 Children of RSV‐associated Hospitalizations Among Children < 5 years of Age Based on Five Methods, Hamilton County, Ohio, 2009–2017.
**Table S2.** Number of RSV‐specific, Unspecified Pneumonia, and Unspecified Bronchiolitis ICD9/10 Coded Hospitalizations from November to April in Hamilton County, Ohio Children Aged < 5 years and Estimated Incidence of RSV‐associated hospitalization per 1000 Children (2009–2017).
**Table S3:** Reasons patients were missed by one surveillance system.
**Table S4.** ICD codes included in analysis.
**Table S5.** Overall average enrollment days, eligibility criteria, and exclusion criteria for the active surveillance systems, both pre 2015 (CCHMC led surveillance) and post 2015 (NVSN surveillance). ^†^

## Data Availability

The data that support the findings of this study are available on request from the corresponding author. The data are not publicly available due to privacy or ethical restrictions.

## Endnotes

^1^
{((a + c + 1)*(a + b + 1))/(a + 0.5) − 1.5}, where a = the number captured by both systems, b = the number captured by the active system alone, and c = the number captured by the passive system alone.

^2^
In cases where ICD10 codes were retroactively added to earlier hospitalizations, ICD10 codes were used. ICD10 codes were exclusively used after December 2016.

^3^
See, for example, 45 C.F.R. part 46, 21 C.F.R. part 56; 42 U.S.C. §241(d); 5 U.S.C. §552a; 44 U.S.C. §3501 et seq.
