# Supplementary material for: Using Multiple Methods to Estimate Respiratory Syncytial Virus (RSV)‐associated Hospitalization Rates in Children Aged < 5 Years—Hamilton County, Ohio, 2009–2017
Source: Influenza Other Respir Viruses. 2025 Apr 16;19(4):e70096. doi: 10.1111/irv.70096 (PMC12000681; doi:10.1111/irv.70096)
Supplement: Supplementary file 1 — Table S1. Estimated Number and Rate per 1000 Children of RSV‐associated Hospitalizations Among Children < 5 years of Age Based on Five Methods, Hamilton County, Ohio, 2009–2017. Table S2. Number of RSV‐specific, Unspecified Pneumonia, and Unspecified Bronchiolitis ICD9/10 Coded Hospitalizations from November to April in Hamilton County, Ohio Children Aged < 5 years and Estimated Incidence of RSV‐associated hospitalization per 1000 Children (2009–2017). Table S3: Reasons patients were missed by one surveillance system. Table S4. ICD codes included in analysis. Table S5. Overall average enrollment days, eligibility criteria, and exclusion criteria for the active surveillance systems, both pre 2015 (CCHMC led surveillance) and post 2015 (NVSN surveillance). † [file IRV-19-e70096-s001.docx]

| **Supplemental Table 1.** Estimated Number and Rate per 1,000 Children of RSV-associated Hospitalizations Among Children <5 years of Age Based on Five Methods, Hamilton County, Ohio, 2009–2017 | | | | | | | | | | |
| --- | --- | --- | --- | --- | --- | --- | --- | --- | --- | --- |
|  | **Stratified** **Capture-Recapture**^†^ | | **Crude Capture-Recapture** | | **Adjusted Active**^‡^ | | **Passive** | | **ICD 9/10 Coding** | |
| **Surveillance Year** | **Rate** | **95% CI**^§^ | **Rate** | **95% CI**^§^ | **Rate** | **95% CI**^§^ | **Rate** | **95% CI**^¶^ | **Rate** | **95% CI**^§^ |
| 2009–10 | 3.5 | (2.3, 6.2) | 7.5 | (4.6, 15.3) | 2.1 | (1.2, 3.2) | 1.6 | (1.3, 2.0) | 4.5 | (4.1, 4.9) |
| 2010–11 | 4.4 | (3.4, 6.5) | 8.6 | (6.2, 13.4) | 3.3 | (2.0, 5.1) | 2.2 | (1.8, 2.7) | 4.4 | (4.0, 4.8) |
| 2011–12 | 4.8 | (3.2, 7.6) | 7.3 | (4.4, 15.0) | 1.5 | (.8, 2.8) | 1.4 | (1.1, 1.7) | 3.7 | (3.4, 4.1) |
| 2012–13 | 5.4 | (4.3, 7.5) | 10.9 | (8.0, 15.8) | 5.6 | (3.3, 9.2) | 2.4 | (2.0, 2.8) | 5.3 | (4.8, 5.7) |
| 2013–14 | 5.5 | (4.1, 8.0) | 7.8 | (5.7, 12.1) | 3.7 | (2.5, 5.2) | 1.6 | (1.3, 2.0) | 3.7 | (3.3, 4.1) |
| 2014–15 | 5.1 | (4.5, 5.8) | 6.7 | (5.8, 7.9) | 7.1 | (4.7, 10.0) | 2.1 | (1.8, 2.6) | 4.9 | (4.5, 5.4) |
| 2015–16 | 5.8 | (4.8, 7.3) | 7.1 | (5.6, 9.5) | 5.1 | (3.4, 7.6) | 1.6 | (1.3, 2.0) | 4.0 | (3.6, 4.3) |
| 2016–17 | 5.3 | (3.6, 8.9) | 7.8 | (5.0, 14.7) | 3.7 | (2.3, 5.8) | 0.8 | (0.6, 1.1) | 3.2 | (2.9, 3.5) |
| Overall | 5.0 | (3.8, 7.2) | 7.9 | (5.7, 13.0) | 4.0 | (2.5, 6.1) | 1.7 | (2.1, 4.4) | 4.2 | (3.8, 4.6) |
| ^†^Analysis limited to those admitted on enrollment days. Analysis stratified by variables that may have influenced the probability of capture in one or both systems (race, age, and ICU status) then numbers of hospitalizations from all strata were totaled to estimate the overall RSV-associated hospitalization rates. Analysis used Evans’ small sample size adjustment. | | | | | | | | | | |
| ^‡^Adjusted for number of days of surveillance and proportion enrolled | | | | | | | | | | |
| ^§^Confidence intervals calculated using 10,000 bootstrap replicates and using adjusted bootstrap percentile method | | | | | | | | | | |
| ^¶^Poisson confidence intervals | | | | | | | | | | |

**Supplemental Tables:**

| **Supplemental Table 2**. Number of RSV-specific, Unspecified Pneumonia, and Unspecified Bronchiolitis ICD9/10 Coded Hospitalizations from November to April in Hamilton County, Ohio Children Aged <5 years and Estimated Incidence of RSV-associated hospitalization per 1,000 Children (2009-2017) | | | | | | |
| --- | --- | --- | --- | --- | --- | --- |
| **Surveillance** **Year** | **RSV-specific (N)** | **Unspecified Pneumonia (N)** | **Unspecified Bronchiolitis (N)** | **Estimated Number of Hospitalizations**† | **Population**^‡^ | **Rate per 1,000** (**95% CI**^§^**)** |
| 2009–10 | 78 | 223 | 379 | 239.1 | 53,047 | 4.5 (4.1, 4.9) |
| 2010–11 | 101 | 175 | 325 | 233.5 | 53,187 | 4.4 (4.0, 4.8) |
| 2011–12 | 68 | 160 | 334 | 200.2 | 53,378 | 3.7 (3.4, 4.1) |
| 2012–13 | 127 | 158 | 406 | 280.6 | 53,306 | 5.3 (4.8, 5.7) |
| 2013–14 | 87 | 124 | 288 | 198.2 | 53,415 | 3.7 (3.3, 4.1) |
| 2014–15 | 118 | 175 | 366 | 263.0 | 53,592 | 4.9 (4.5, 5.4) |
| 2015–16 | 86 | 121 | 334 | 214.0 | 54,192 | 4.0 (3.6, 4.3) |
| 2016–17 | 42 | 117 | 341 | 171.7 | 53,694 | 3.2 (2.9,3.5) |
| Overall | 707 | 1,253 | 2,773 | 1,800.3 | 427,811 | 4.2 (3.8, 4.6) |
| †Estimated hospitalizations used all RSV-specific coded hospitalizations as well as 20% of unspecified pneumonia and 30% of unspecified bronchiolitis codes from patients admitted from November to April  ^‡^US census estimates of children aged less than five years in Hamilton County, Ohio  ^§^Confidence intervals calculated using 10,000 bootstrap replicates and using adjusted bootstrap percentile method | | | | | | |

| **Supplemental Table 3:** Reasons patients were missed by one surveillance system | |
| --- | --- |
| **Patients Missed by Passive Surveillance (N=520)** | **n (%)** |
| Not Tested | 505 (97) |
| Tested; RSV Negative | 15 (3) |
| **Patients Missed by Active Surveillance (N=583)** | **n (%)** |
| Non-surveillance day | 324 (56) |
| Eligible; not enrolled | 107 (18) |
| No parent or guardian present | 48 (45) |
| Interpreter needed | 19 (18) |
| Refused | 40 (37) |
| Missed (never screened) | 75 (13) |
| Not eligible for enrollment | 41 (7) |
| Enrolled and tested; RSV– | 36 (6) |

| **Supplementary Table 4.** ICD codes included in analysis. | | |
| --- | --- | --- |
| **Category** | **ICD-9** | **ICD-10** |
| RSV-specific | 466.11, 480.1, 079.6 | J210, B974, J121, J205 |
| Unspecified Bronchiolitis | 466.1 (excluding 466.11) | J218, J219 |
| Pneumonia not coded as RSV | 480–486 (excluding 480.1) | J12 (excluding J121), J13, J14, J15, J16, J17, J18 (excluding J182), A481, B250, A3791, A221, B440, A3701, A3711, A3781, A3791, B7781 |

| **Supplemental Table 5**. Overall average enrollment days, eligibility criteria, and exclusion criteria for the active surveillance systems, both pre 2015 (CCHMC led surveillance) and post 2015 (NVSN surveillance). ^†^ | |
| --- | --- |
| **<2015 (CCHMC Only Active Surveillance)** | **>2015 (NVSN Active Surveillance)** |
| **Enrollment** | |
| 3.25 days a week | 4.8 days a week |
| **Eligibility Criteria** | |
| < 5 years of age | < 18 years of age (only those <5 used in analysis |
| Reside in Hamilton County | Reside in a surveillance site area (Hamilton County for CCHMC) |
| Presented with fever and/or symptoms of an acute respiratory infection | Presented with fever and/or symptoms of an acute respiratory infection |
| Admitted to hospital within 48 hours of enrollment | Admitted to hospital within 48 hours of enrollment |
| One or more of the following admission diagnoses: acute respiratory illness, apnea, asthma exacerbation, bronchiolitis, croup, cystic fibrosis exacerbation, RSV febrile neonate, febrile seizure, influenza, fever without localizing signs, respiratory distress, pneumonia/ pneumonitis, rule out sepsis, sinusitis, tonsillitis, pharyngitis strep throat, otitis media or upper respiratory infection |  |
|  | Illness duration of < 14 days |
| **Exclusion Criteria** | |
| Neutropenia | Neutropenia |
| Newborn who had never been discharged from hospital | Newborn who had never been discharged from hospital |
| Children whose parents or guardians were unable to understand the consent process, including those with a language barrier |  |
| Enrolled as outpatients within the previous 6 days |  |
| Enrolled as ED patients within the previous 4 days |  |
|  | Known non-respiratory cause for hospitalization |
|  | Admitted <5 days after a previous hospitalization |
|  | Transferred from another hospital after an admission of >48 hours |
|  | Previously enrolled in study <14 days before current hospitalization |
| ^†^Inclusion in analysis was adjusted to reconcile differences in eligibility and exclusion criteria. However, it is possible that differences may have affected which patients were captured by surveillance year | |
